# When Ascites Is Not Ascites!

**DOI:** 10.7759/cureus.60868

**Published:** 2024-05-22

**Authors:** Mei Yang, Aboud Fahel, Thomas Pohlman, Ravi Donepudi, Sajid Zafar

**Affiliations:** 1 Internal Medicine, St. Luke's Hospital, Chesterfield, USA; 2 Gastroenterology and Hepatology, St. Luke's Hospital, Chesterfield, USA

**Keywords:** visceral artery, visceral artery rupture, visceral artery aneurysms, splenic artery aneurysm, embolization, hemorrhagic shock, splenic artery, ruptured, aneurysm, ascites

## Abstract

Patients presenting with ascites should be properly evaluated to differentiate potential etiologies. Then, based on the evaluation, we can tailor more accurate treatment plans for patients. Cirrhosis is the most common cause, and others include cancer, heart failure, and, in our case, rarely a visceral artery rupture. Rupture of the splenic artery aneurysm can be lethal and should be considered as a possible differential in a patient with no previous history of heart failure, cancer, or cirrhosis. Our patient was identified after an initial misdiagnosis of possible ascites secondary to cirrhosis. However, input from an interventional radiologist led to proper identification and tailored management. Early treatment is crucial to prevent complications, including death.

## Introduction

Presenting with ascites and proper evaluation of the etiology is important to tailor the treatment approach. Cirrhosis is the most common cause of ascites, and other causes, including cancer, heart failure, and visceral rupture, are much rarer. Splenic artery aneurysms are more common in women, with a female-to-male ratio of 4:1 [1]. Patients typically present in the sixth or seventh decade of life [2]. The rupture of the splenic artery aneurysm can lead to massive, life-threatening bleeding, resulting in hemodynamic instability. The ruptured splenic artery aneurysm usually leaks into the free peritoneal space and, on occasion, into the gastrointestinal tract. For splenic artery aneurysms, the related etiology can be variable, and the clinical presentation can be the emergence of acute abdominal symptoms or even death if it is ruptured. Computed tomography (CT), magnetic resonance imaging (MRI), and Doppler ultrasound are the most commonly used to locate the splenic artery aneurysm. Treatment options range from endovascular approaches to surgical interventions, including splenectomy. We report a case of a patient with two splenic artery aneurysms where one ruptured into the free peritoneal space, resulting in hemorrhagic shock, which was successfully treated with emergency embolization. 

## Case presentation

We present a case of a 71-year-old Caucasian female with a past medical history known for atrial fibrillation (on Apixaban), hypertension (on Metoprolol), urinary tract infection (UTI), chronic back pain, and lower extremity lymphedema. The patient had presented to the emergency department (ER) with generalized weakness, abdominal pain, and near syncope for the past week. Regarding her past family history, her mother had hypertension and an aortic aneurysm. On physical examination, her abdomen was soft and non-tender to touch. Abdominal distension was consistent with signs of mild-moderate ascites. In the ER, she was noted to have elevated lactic acid and leukocytosis on lab tests. A CT of the abdomen and pelvis showed features of hepatic cirrhosis and a moderate amount of ascites. It also showed a right adnexal calcified lesion most likely of ovarian origin, right renal focal scarring, a tiny calculus, left colonic diverticulosis, basilar interstitial infiltrates, and pleural effusions. She was admitted under the impression of atrial fibrillation with rapid ventricular response, cirrhosis with ascites, and near syncope. The patient was admitted to the floor with a consult requested from gastroenterology and cardiology. There was no prior liver disease or history of alcohol abuse.

**Figure 1:**
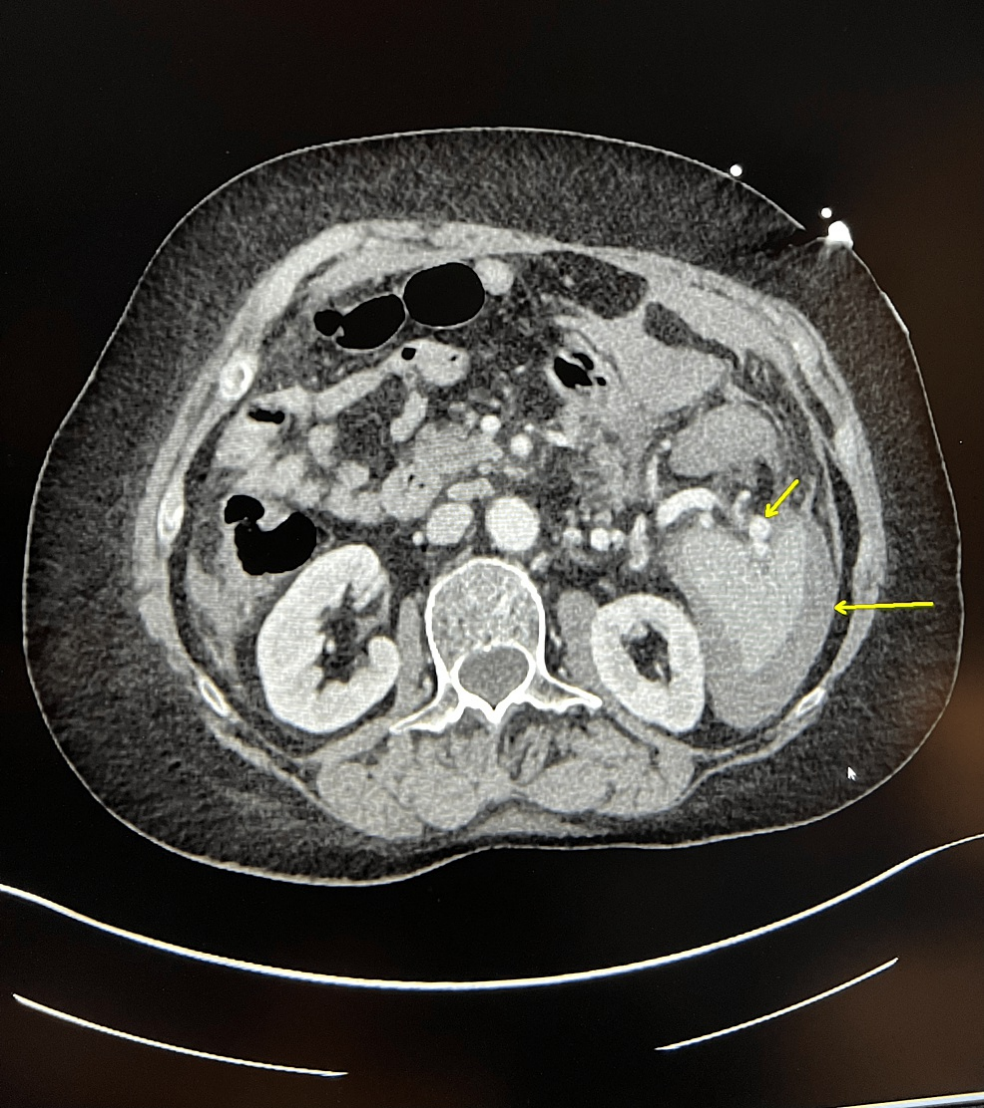
Splenic artery aneurysm (short arrow) and hemorrhage (long arrow)

**Figure 2:**
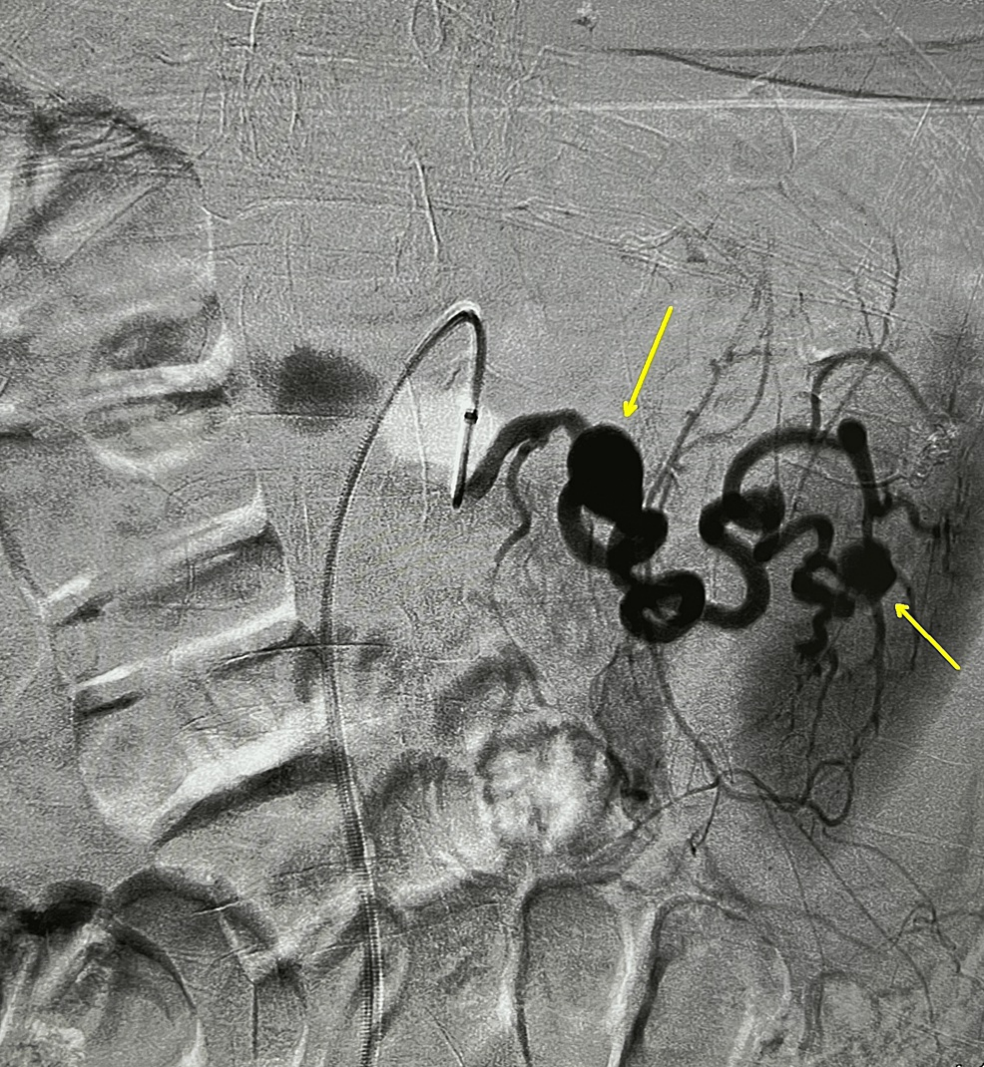
Angiogram showing two splenic artery aneurysms. The right arrow is the ruptured splenic artery aneurysm

**Figure 3:**
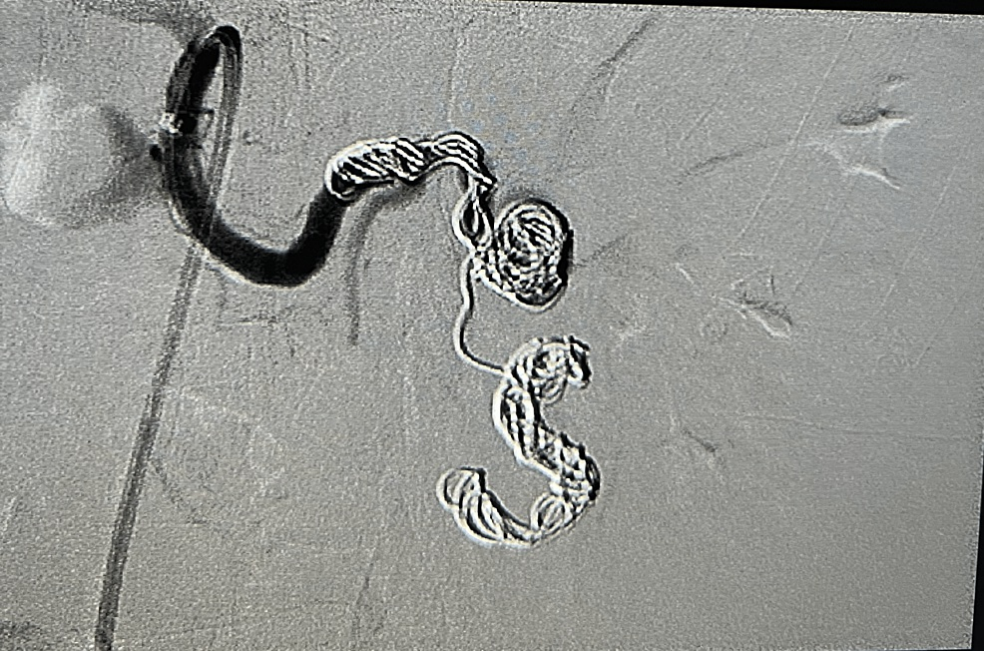
Statue-post coiling of the splenic artery aneurysm

On the second day of admission, the patient’s blood pressure dropped to 78/47 mmHg, and she complained of diffuse abdominal pain, lightheadedness, dizziness, and shortness of breath while on 6 L of nasal cannula oxygen. The patient was treated with ceftriaxone for suspected sepsis and spontaneous bacterial peritonitis, with a component of third spacing in the setting of cirrhosis and ascites. The gastroenterology team advised abdominal paracentesis due to the results of the imaging. Just before abdominal paracentesis, the interventional radiologist noticed an acute rupture of the splenic artery aneurysm with a large volume of hemoperitoneum on imaging (Figure 1), and then a shift of diagnosis occurred from initially being ascites secondary to cirrhosis to a hemoperitoneum secondary to ruptured splenic artery aneurysm. The patient was then rushed for an emergent splenic artery embolization. In the interventional radiology department, an ultrasound evaluation revealed a widely patent right common femoral artery. The aortogram revealed a normal-caliber aorta. The superior mesenteric artery is widely patent, while the celiac, left gastric, and common hepatic arteries are patent. The splenic artery was tortuous. There was a large mid-splenic artery aneurysm as well as an aneurysm in the splenic hilum. The aneurysm in the splenic hilum was seen to be actively extravasating and was at the terminus of subsegmental branches of the splenic artery (Figure 2). Devascularization of the spleen with embospheres was performed, followed by coil embolization across the neck of the larger, more proximal mid-splenic artery. There was stasis in the splenic artery (Figure 3). The patient was transferred to the intensive care unit (ICU) for closer monitoring. Elastography was also done at the bedside and did not show significant fibrosis to suggest cirrhosis of the liver. The patient was kept overnight in the ICU and then was stable enough to be transferred to the floor for further management. 

## Discussion

It is important to have an approach to evaluating newly diagnosed ascites. Cirrhosis is the most common cause of ascites, and that was our first differential. Cirrhosis accounts for 81% of the cases, followed by cancer at 10%, heart failure at 3%, tuberculosis at 2%, dialysis and pancreatic diseases at 1%, and other causes, including hemoperitoneum, among others, at 2% [3]. It is critical to try to exclude other causes through an extensive history, physical exam, imaging, and ascitic fluid analysis if paracentesis was obtained. In our case, proper assessment by imaging initially failed to detect the etiology, which was then identified by the interventional radiologist. Simple ascites tend to have a density of 0-30 Hounsfield units (HU). It is difficult for CT imaging to differentiate between bile, urine, and serous fluid within the ascites. Chylous has a density of less than 0 HU. As for the hemoperitoneum, it has a density of more than 30 HU [4]. This was a major differentiating factor that could direct your diagnosis to a particular etiology. The splenic artery is defined as aneurysmal when a focal dilation is found in its diameter of greater than 50% compared to the normal vessel diameter, which ranges from 0.43 cm to 0.49 cm [5]. True aneurysms develop secondary to arterial wall weakness from several possible causes, including atherosclerosis (32%), medial degeneration or dysplasia (24%), abdominal trauma (10%), portal hypertension, multiple pregnancies, congenital anomalies, including collage vascular disease, and vasculitis, including polyarteritis nodosa [5-8].

The rate of rupture of splenic artery aneurysms is between 2% and 10% in asymptomatic patients, increasing to 76-83% in symptomatic patients [5,6]. Tessier et al.’s literature review revealed common splenic artery aneurysm cases: chronic pancreatitis (46%), trauma (29%) as the cause in 29, and unknown etiology (14%) of the documented cases [9]. In regard to the case, bedside elastography did not show significant fibrosis to suggest cirrhosis of the liver. Although in the current setting, the finding of the elastography may not be very reliable, this made portal hypertension secondary to liver cirrhosis a less likely cause for this patient’s aneurysmal formation.

Endovascular vessel embolization or splenectomy via emergency laparotomy is the common management of splenic artery aneurysm rupture, which has variable techniques according to the type of aneurysm, location, and condition of the collaterals [5,10]. Some tortuous aneurysms are treated with aneurysmal coiling techniques, like the case we present, which was treated successfully with immediate embolization by coiling [5].

## Conclusions

We present a case of a patient presenting with new ascites and how a shift in diagnosis happened based on imaging. It is crucial to identify the etiology of the ascites to tailor the management. In our case, it was hemoperitoneum secondary to a ruptured splenic artery aneurysm that resulted in a hemorrhagic shock that was successfully treated with immediate embolization. This is a rare cause of ascites that was initially missed and would have been catastrophic if an early diagnosis was not made. Splenic artery aneurysm rupture is a rare but fatal disease and should be considered in patients with high-risk medical illnesses. It is crucial to be aware of this condition to make timely treatment decisions that are critical to saving the patient's life.
